# Usefulness of Elobixibat in Patients With Chronic Constipation After Cholecystectomy

**DOI:** 10.7759/cureus.67132

**Published:** 2024-08-18

**Authors:** Yuji Sakai, Toshio Tsuyuguchi, Junichiro Kumagai, Hiroshi Ohyama, Takashi Kaiho, Masayuki Ohtsuka, Naoya Kato

**Affiliations:** 1 Department of Gastroenterology, Sakai Clinic, Kimitsu, JPN; 2 Department of Gastroenterology, Chiba Prefectural Sawara Hospital, Sawara, JPN; 3 Department of Gastroenterology, Kimitsu Central Hospital, Kisarazu, JPN; 4 Department of Gastroenterology, Graduate School of Medicine, Chiba University, Chiba, JPN; 5 Department of Surgery, Kimitsu Central Hospital, Kisarazu, JPN; 6 Department of General Surgery, Graduate School of Medicine, Chiba University, Chiba, JPN

**Keywords:** post-cholecystectomy, chronic constipation, constipation, elobixibat, bile acid

## Abstract

Background: There have been reports that elobixibat improves bowel movements in patients with chronic constipation. However, no studies have been conducted to date to examine bowel movements after the administration of elobixibat in patients with chronic constipation in terms of the presence or absence of the gallbladder. In this study, we examined the frequency of bowel movements and stool forms in patients with gallbladders and post-cholecystectomy patients before and after the administration of elobixibat for chronic constipation.

Methods: Elobixibat 10 mg was administered to treat chronic constipation in 40 patients with gallbladders and 18 patients who underwent cholecystectomy. The frequencies of bowel movements one week before and after elobixibat administration were compared between the two groups, using the Bristol Stool Form Scale (BSFS).

Results: No significant difference in patient background with or without cholecystectomy was noted between the groups. In patients with gallbladders, the pre-dosing mean frequency of bowel movements was 2.389 ± 0.502 with BSFS of 2.179 ± 0.721 and the post-dosing mean frequency of bowel movements was 4.308 ± 1.151 with BSFS of 3.718 ± 1.521, indicating significant improvement in bowel movements (p < 0.001). In post-cholecystectomy patients, the pre-dosing mean frequency of bowel movements was 2.389 ± 0.502 with BSFS of 2.222 ± 0.647 and the post-dosing mean frequency of bowel movements was 4.222 ± 1.734 with BSFS of 3.333±1.237, indicating significant improvement in bowel movements (p < 0.001). No significant difference in bowel movements was noted between patients with or without the gallbladder.

Conclusions: Elobixibat is useful in improving the bowel movements of patients with chronic constipation. No significant difference was noted in the improvement of bowel movements due to cholecystectomy. It was suggested that even post-cholecystectomy patients could obtain therapeutic effects similar to patients with gallbladders.

## Introduction

It has been reported that the quality of life (QOL) in patients with chronic constipation is undermined as compared with healthy adults [1-5]. Furthermore, chronic constipation may affect a long-term prognosis as it is reported to raise risks for cardiovascular diseases/deaths and to be involved in the onset of Parkinson’s disease [6-8]. Against this backdrop, we find it extremely important to improve and resolve chronic constipation. There have been reports that diet therapy of eating fruits and meals containing lots of fiber is useful [9,10]. The usefulness of exercise therapy has also been reported as evidenced by aerobic exercises improving constipation symptoms [11]. For patients whose symptoms fail to improve after all these attempts, pharmacotherapy will be considered. In pharmacotherapy, drugs with various mechanisms of action are available, and their usefulness has been reported [12-16]. Stimulative laxatives, osmotic laxatives, and other drugs have been used for patients with chronic constipation.

Elobixibat is a therapeutic drug with a new mechanism of action. Elobixibat inhibits an ileal bile acid transporter (IBAT) inhibitor, to induce aqueous secretion in the large intestine through the action of bile acid that flows in from the large intestine, and further promotes the large intestine movement. These two actions promote bowel movements and improve stool forms and QOL [17,18]. Bile acid is the main ingredient of bile. When the gallbladder is present, bile is stored in the gallbladder, but after removal of the gallbladder, bile is said to flow continuously into the duodenum [19]. Thus, elobixibat may have different effects depending on the presence or absence of the gallbladder, which is involved in the outflow of bile into the duodenum. Elobixibat is a useful drug for chronic constipation, but because it is a new drug, there have been few reports of its use.

As far as we know, no report to date has studied the efficacy of elobixibat in chronic constipation of patients with or without gallbladders. At this time, we report our study on whether the effect of elobixibat differs depending on the presence or absence of the gallbladder in patients with chronic constipation.

## Materials and methods

This study targeted patients with chronic constipation who were prescribed elobixibat at Sakai Clinic after it became available to patients in Japan. The subjects of our study were 58 patients diagnosed with chronic constipation at Sakai Clinic from May 2018 to March 2024. Rome IV Criteria were used to diagnose chronic constipation [20]. Patients who regularly visit the outpatient clinic were selected. Subjects who met the following inclusion and exclusion criteria were eligible for inclusion. Inclusion criteria were (i) patients diagnosed with chronic constipation based on the Rome IV criteria; (ii) patients who are 20 years old or older at the time of diagnosis; (iii) patients who can come to the hospital as outpatients; and (iv) patients who understood the study well and have given the consent after receiving enough explanation for participating in this clinical study. Exclusion criteria were (i) patients diagnosed with temporary constipation; (ii) patients with extremely poor general condition; (iii) patients whom the study doctor considered to be ineligible as the subject; and (iv) patients who have an allergy to drugs. The drug was administered to all patients with chronic constipation who were considered eligible for the drug, regardless of whether or not they were receiving oral medication for chronic constipation. Elobixibat was administered at a dose of 10 mg once daily before the evening meal. There were 40 patients with gallbladders and 18 post-cholecystectomy patients. No ileal resection patient was found in these patients. Stool forms were assessed, using the Bristol Stool Form Scale (BSFS) [21].

Data in this study at Sakai Clinic were used after obtaining prior consent from all patients. For patients who had been administered elobixibat, we checked in the outpatient clinic to see if the data could be used, and after obtaining their consent, we conducted the analysis. Because Sakai Clinic does not have an ethics committee, we asked Kimitsu Central Hospital to conduct an ethical review of this study. This study was approved by the Ethical Committee of Kimitsu Central Hospital (IRB number 775). This study is a retrospective observational study.

Statistical analysis

The paired t-test or Wilcoxon signed-rank test was performed to compare data within each group before and after the administration of elobixibat. Binary variables were compared using the χ2 test. In all cases, a p-value <0.05 was regarded as statistically significant. Data were analyzed using SPSS software version 17 (released 2008, SPSS, Chicago, IL). Data are shown as means and standard deviations (SDs), unless otherwise stated. The differences between the two groups receiving elobixibat were calculated as p-values ​​using the F-test. The p-values ​​of the F-test were significant at the two-sided significance level of 5%.

## Results

The patient background is summarized in Table 1. 

**Table 1:** Patient list SD: standard deviation

Number of patients	58
With cholecystectomy, (%)	18 (31)
Sex, No. (%)	
Male/Female	20 (34.5)/38 (65.5)
Age, (± SD)	66.625 ± 13.27
Bristol Stool Form Scale, No. (%)	
Type 1	11 (19)
Type 2	25 (43.1)
Type 3	22 (37.9)
Type 4	0 (0)
Type 5	0 (0)
Type 6	0 (0)
Type 7	0 (0)
One-week frequency of bowel movements before elobixibat administration (± SD)	2.386 ± 0.526
The number of drugs to relieve constipation before elobixibat administration, No. (%)	
0	18 (31)
1 Magnesium oxide tablets,	30 (51.7)
2 Magnesium oxide tablets + sennoside	10 (17.2)
Comorbidity, No. (%)	
Hypertension	28 (48.3)
Hyperlipidemia	26 (44.8)
Diabetes mellitus	25 (43.1)
Cerebrovascular disease	8 (13.8)
Chronic respiratory disease	8 (13.8)
Cardiac disease	16 (27.6)
Chronic liver disease	16 (27.6)
Chronic renal disease	3 (5.2)
Malignant disease	6 (10.3)

There were 20 men and 38 women, and the mean (±SD) age of the patients in the study was 66.625 (±13.27). Stools assessed in the BSFS were type 1 (11 cases), type 2 (25 cases), type 3 (22 cases), type 4 (0 cases), type 5 (0 cases), type 6 (0 cases), and type 7 (0 cases). The mean frequency of bowel movements one week before elobixibat administration was 2.386 (±0.526). The number of drugs used to relieve chronic constipation before elobixibat administration was 0 in 18 patients, one in 30 patients, and two in 10 patients. Comorbidities were hypertension in 28 patients (48.3%), hyperlipidemia in 26 patients (44.8%), diabetes mellitus in 26 patients (44.8%), cerebrovascular disease in eight patients (13.8%), chronic respiratory disease in eight patients (13.8%), heart disorder in 16 patients (27.6%), chronic liver disease in 16 patients (27.6%), chronic renal disease in three patients (5.2%), and malignant disease in six patients (10.3%). Elobixibat 10 mg was administered before the evening meal. We examined whether there are any changes in the frequency of bowel movements and stool forms one week before and after elobixibat administration depending on the presence or absence of cholecystectomy. Patient backgrounds by the presence or absence of the gallbladder are summarized in Table 2.

**Table 2:** Comparison of patient background by the presence or absence of cholecystectomy SD: standard deviation

	Without cholecystectomy	With cholecystectomy	P-value
Number of patients, No. (%)	40	18	
Sex, No. (%)			
Male/ Female	12(30) /28(70)	6(33.3)/ 12(66.6)	0.5149
Age, (± SD)	66.200±15.405	67.571±11.566	0.7097
Bristol Stool Form Scale, No. (%)			
Type1	8(20)	3(16.7)	0.5365
Type2	16(40)	9(50)	0.3343
Type3	16(40)	6(33.3)	0.4277
Drugs to relieve constipation, No. (%)			
0	12(30)	6(33.3)	05149
1 magnesium oxide tablets	20(50)	10(55.6)	0.4577
2 magnesium oxide tablets + sennoside	8(20)	2(16.7)	0.1809
Comorbidity, No. (%)			
Hypertension	18(45)	10(55.6)	0.5729
Hyperlipidemia	17(42.5)	9(50)	0.776
Diabetes mellitus	17(42.5)	9(50)	0.776
Cerebrovascular disease	6(15)	2(11.1)	0.5216
Chronic respiratory disease	5(12.5)	3(16.7)	0.3769
Cardiac disease	12(30)	4(22.2)	0.7523
Chronic liver disease	12(30)	4(22.2)	0.7523
Chronic renal disease	2(5)	1(5.6)	0.6798
Malignant disease	4(10)	2(11.1)	0.6125

No significant difference was noted in the patient background depending on the presence or absence of cholecystectomy. When compared one week before and after elobixibat administration, the frequency of bowel movements and stool forms significantly improved (Tables 3, 4).

**Table 3:** Elobixibat changes in bowel movements before and after elobixibat administration BSFS: Bristol Stool Form Scale, SD: standard deviation

Total 58 cases	Comparison of before and after administration	P-value
Elobixibat pre-elobixibat defecation count/week, (± SD)	2.386 ± 0.526	
Elobixibat post-elobixibat defecation count/week, (± SD)	4.281 ± 1.346	<0.001
Elobixibat pre-elobixibat BSFS, (± SD)	2.2105 ± 0.7255	
Elobixibat post-elobixibat BSFS, (± SD)	3.4561 ± 1.3896	<0.001

**Table 4:** Comparison of changes in bowel movements before and after elobixibat administration by the presence or absence of cholecystectomy BSFS: Bristol Stool Form Scale, SD: standard deviation

	Without cholecystectomy	With cholecystectomy	P-value	Comparison of before and after elobixibat administration
Elobixibat pre-elobixibat defecation count/week, (± SD)	2.385 ± 0.544	2.389 ± 0.502	0.6455	
Elobixibat post-elobixibat defecation count/week, (± SD)	4.308 ± 1.151	3.718 ± 1.521	0.3618	<0.001
Elobixibat pre-elobixibat BSFS, (± SD)	2.179 ± 0.721	2.222 ± 0.647	0.978	
Elobixibat post-elobixibat BSFS, (± SD)	3.718 ± 1.521	3.333 ± 1.237	0.826	<0.001

No significant difference in improvement attributable to elobixibat administration was noted in the frequency of bowel movements and stool forms of patients with or without gallbladders (Table 4). Adverse events caused by elobixibat administration were noted in 11 patients (19%): abdominal pain in six patients (10.3%) and diarrhea in five patients (8.6%). One patient (1.7%) discontinued the oral treatment of elobixibat on day 3 of the administration due to frequent diarrhea. The rest of the adverse events were negligible and conservatively resolving, and the oral treatment was continued. No significant difference was noted in the incidence of adverse events due to elobixibat administration in patients with gallbladders and post-cholecystectomy patients (Table 5).

**Table 5:** Comparison of adverse events to elobixibat by the presence or absence of cholecystectomy

	Without cholecystectomy	With cholecystectomy	P-value
Number of patients	40	18	
Elobixibat adverse events after elobixibat administration of elobixibat, No. (%)			
Abdominal pain	4(10)	2(11.1)	0.5149
Diarrhea	4(10)	1(5.6)	0.5026
Total	8(20)	3(16.7)	0.5365

## Discussion

In chronic constipation, the decreased bile acid level in stool is reported, indicating a close link between the bile acid and the pathological condition of constipation [17]. Bile acid is a main ingredient of bile and biosynthesized by cholesterol in the liver. A large part of the bile acid is reabsorbed by IBAT at the terminal ileum and used by the liver. The main roles of bile acid are to adjust cholesterol in the body and to digest and absorb the lipids in the small intestines. When the bile acid not reabsorbed by IBAT flows into the large intestine, it acts on a transmembrane G protein-coupled receptor present on intestinal epithelial cells, activates a cystic fibrosis transmembrane conductance regulator via cAMP, and promotes aqueous secretion by inducing the secretion of C1 ions into the intestinal tract. Bile acid also releases 5-hydroxytryptamine through the transmembrane G protein-coupled receptor of enterochromaffin cells to promote peristaltic movement of the large intestine [22]. Elobixibat inhibits the reabsorption of bile by restraining IBAT at the terminal ileum from increasing the amount of bile acid in the large intestine. As a result, bile acid acts to promote aqueous secretion and peristaltic movement inside the intestinal tract, thereby increasing bowel movements and improving stool forms as reported in past studies [17,18]. It is also reported that the first spontaneous bowel movement induced by the administration was marked at 25.5 hours in the placebo group and 5.2 hours in the elobixibat group, indicating significant early bowel movement resulted from the use of elobixibat [18].

In our study, elobixibat administration also resulted in increased bowel movements and improved stool forms in patients with chronic constipation. In view of the major impact of bile on the action of this drug, we examined the usefulness of elobixibat in terms of the presence or absence of the gallbladder. In this study, elobixibat improved bowel movements and stool forms of chronic constipation in post-cholecystectomy patients similar to those of patients with gallbladders. The gallbladder acts to retain and concentrate the bile. Meal intake contracts the gallbladder, creating an outflow of bile into the duodenum [23]. Elobixibat is generally taken before meals [24]. Postprandial administration makes it difficult for a large quantity of bile to flow into the duodenum because the gallbladder is in a contracted state. Patients without gallbladders also retain bile as it flows out from the duodenal papilla; they just do not have a place to concentrate bile as those with gallbladders do. It would have been possible for elobixibat to properly improve the frequency of bowel movements and stool forms.

The effectiveness of this drug cannot be expected in patients with decreased hepatic function such as child C with hepatic cirrhosis or those with obstructive jaundice. In this study, we experienced a patient with jaundice at a visit one week after elobixibat administration. Abdominal ultrasound conducted then revealed that the patient had experienced obstructive jaundice. No jaundice was noted before the administration. Jaundice was obstructive jaundice caused by a fall of a gallbladder stone into the common bile duct. In fact, this patient showed no changes in the frequency of bowel movements and stool form (twice, BSFS type 1) one week before and after elobixibat administration. When the outflow of bile into the duodenum is difficult, such as when there are problems in liver parenchyma or biliary obstruction, it is likely that the effectiveness of elobixibat cannot be expected. Generally, if a biliary obstruction is present, drainage is performed to unblock the biliary obstruction. In the case of drainage through the stent placement to enable a proper outflow of bile into the duodenum, the effectiveness of elobixibat can be expected. However, in the case of endoscopic nasobiliary drainage to drain bile outside the body, the effectiveness of elobixibat cannot be expected even if the obstruction is relieved. In patients with a history of ileectomy, who were not present in our study, the effectiveness of elobixibat is less likely to be experienced since IBAT is expressed at the terminal ileum. Adverse events were noted in 11 cases (19%) of these patients. In the past reports, no serious adverse events including death were noted although abdominal pain was reported in many cases [12]. In our study, abdominal pain was reported in six patients (10.3%) and diarrhea in five patients (8.6%), but no other adverse events were reported. Only one patient (1.7%) discontinued elobixibat due to frequent diarrhea symptoms. Other adverse events were negligible and not serious. There have been few reports of serious complications in previous studies [18,24]. This suggests that elobixibat may be relatively safe to use.

This study poses some problems in that it has a small sample size; it is a retrospective study in a single facility where the two groups, with or without the gallbladder, were not compared in a similar number of patients; and it is a study analyzing results only from one week after elobixibat administration. Treatment drugs to relieve constipation may entail a risk for drug resistance after long-term use, and thus, consideration of a long-term prognosis is extremely important [25]. Going forward, a multicenter randomized trial including a long-term prognosis would be necessary. However, even if further studies were to be conducted, the problem is that although there are many patients with chronic constipation, it is difficult to gather a similar number of patients with chronic constipation who have undergone cholecystectomy. This will also be a major challenge. Because colonoscopy was not performed on all cases in advance, there is a possibility that colorectal cancer may have been present. This confirmation may have been necessary. It is still unknown as to which type of chronic constipation conditions elobixibat is more useful, or what type of drugs should be co-administered with elobixibat to deliver more effectiveness. It is essential that we continue this discussion going forward.

## Conclusions

It is useful to administer elobixibat to improve bowel movements in patients with chronic constipation. Elobixibat is also useful in improving symptoms in patients with chronic constipation after cholecystectomy. There have been no serious adverse events associated with the administration of elobixibat, and it is believed to be a relatively safe drug to use.
